# Revisiting entinostat as an immune-potentiating adjuvant

**DOI:** 10.18632/oncotarget.26453

**Published:** 2018-12-18

**Authors:** Tyler R. McCaw, Troy D. Randall, Rebecca C. Arend

**Affiliations:** Tyler R. McCaw: Department of Medicine, Division of Clinical Immunology and Rheumatology, University of Alabama at Birmingham, Birmingham, AL, USA

**Keywords:** entinostat, histone deacetylase inhibitor, immunotherapy, adjuvant, ovarian cancer

Relatively infrequent but profound responses to checkpoint blockade antibodies allude to the paradigm-shifting potential of tumor immunotherapy. Effective engagement of the immune response is a particularly appealing approach to treatment of ovarian cancer (OVCA), as continual T cell surveillance and ability to establish long-lasting memory may be able to combat two recalcitrant features of this disease—metastatic spread throughout the peritoneum and predictable recurrence. It is unfortunate, then, that checkpoint blockade yet benefits only a minority of patients [1]. This raises the question: how can we stimulate and enhance an endogenous tumor-specific immune response in all patients?

Using an orthotopic murine model of OVCA, Smith et al. recently demonstrated that epigenetic manipulation can substantially improve immune-mediated tumor control *in vivo* [2]. Entinostat, the class I histone deacetylase (HDAC) inhibitor used, broadly affects cellular transcription programs (both up and down regulating gene expression), while also altering the biologic activity of over 1750 non-histone proteins by impacting their acetylation status [3]. Given the rather non-specific nature of HDAC inhibition, Smith and colleagues’ analysis of both tumor and responding T cells following entinostat treatment provides useful insight to the tumor-immune dynamic. In this manner, HDAC inhibition led to increased expression of the T cell chemokine CXCL10, recruiting more T cells to the tumor site, as well as upregulation of major histocompatibility complex (MHC) class I and II molecules, providing stronger stimulatory signals to tumor-specific T cells once in the tumor. Increased granzyme B and interferon γ (IFNγ) transcripts found in the tumor of entinostat treated mice would seem to corroborate this notion, suggesting superior effector T cell functions. It is tempting to speculate that entinostat may make certain loci more permissive to IFNγ signaling, leading to the strong upregulation of IFNγ-responsive genes observed in the tumor microenvironment (TME). To this point, when the experiment was repeated using Rag knockout mice (lacking T and B cells) tumor growth was largely rescued and the IFNγ-responsive molecules MHCII and PD-L1 were no longer upregulated. Thus, in this model, both entinostat and adaptive immunity-derived factors, like IFNγ produced by tumor-specific T cells, are required to initiate an inflammatory repolarization of the TME yielding improved control of tumor growth.

This encouraging study by Smith et al. showing robust promotion of an endogenous anti-tumor immune response leads us to consider how entinostat might be incorporated into clinical treatment strategies. Although the drug has not yet demonstrated efficacy as a monotherapy in clinical trials, it may be exceptionally well-suited as an adjuvant therapy in immune-modulating combinatorial strategies. Taking the example of checkpoint blockade: response to anti-PD1 correlates with a pre-existing anti-tumor immune response—T cell infiltration into the tumor and expression of PD-L1 in the TME, which increases in response to IFNγ produced by T and NK cells [4]. Accordingly, entinostat might stimulate sufficient T cell activation and infiltration to then enable response to checkpoint blockade in patients who otherwise would not experience a clinical benefit.

Beyond checkpoint blockade, entinostat’s partnership with adaptive immunity may be amenable to combination with other immunotherapeutic approaches. Smith et al. identified functional changes in both tumor cells and responding T cells following entinostat treatment, thereby targeting multiple aspects of the tumor-immune dynamic using a single agent. Blocking TGFβ signaling might also favorably target multiple components of this dynamic. Specifically, TGFβ promotes OVCA progression through multiple mechanisms [5] and is enriched in the tumors of patients with poor prognoses [6]. Relative to adaptive immunity, TGFβ secretion by regulatory T cells and myeloid derived suppressor cells (among other cellular populations within the TME) impairs T cell proliferation and effector functions and also contributes to exclusion of T cells from the tumor bed [7], severely curtailing their ability to control tumor growth. It follows that combination of entinostat with TGFβ neutralizing antibodies might synergistically benefit the evolving immune response, the former increasing T cell recruitment and tumor immunogenicity, while the latter facilitates T cell ingress into a less suppressive TME.

The findings reported by Smith et al. are an exciting stride toward more efficacious cancer immunotherapy. Specifically, the ability of entinostat to stimulate endogenous anti-tumor immunity may represent a strategy to increase response rates to checkpoint blockade, or less conventional agents like anti-TGFβ, unleashing the potential thereof. Future studies might also investigate the potential to incorporate such combinations in the upfront setting of OVCA. Here, platinum-based chemotherapy, a standard of care, can in fact promote T cell-mediated tumor killing [8] and therefore may afford a convenient opportunity to initiate entinostat-based combinatorial regimens. In order for entinostat to realize its clinical potential, however, careful consideration must be given to both the sequence and timing in which it is integrated in combinatorial strategies such that a potential synergistic relationship between epigenetic- and immune-based approaches can be offered as a new treatment avenue for OVCA patients.
